# The metazoan parasite community of the barred grunt *Conodon nobilis* (Actinopterygii: Haemulidae) from the coast off Rio de Janeiro, southeastern Brazil

**DOI:** 10.1590/S1984-29612024068

**Published:** 2024-11-22

**Authors:** Fabiano Paschoal, Jorge Luiz Silva Nunes, Anderson Dias Cezar, Felipe Bisaggio Pereira, Jose Luis Luque

**Affiliations:** 1 Departamento de Microbiologia, Imunologia e Parasitologia, Universidade Do Estado Do Rio de Janeiro – UERJ, Rio de Janeiro, RJ, Brasil; 2 Laboratório de Organismos Aquáticos, Universidade Federal do Maranhão – UFMA, São Luís, MA, Brasil; 3 Departamento de Pós-graduação e Pesquisa, Faculdade Presbiteriana Mackenzie Rio, Rio de Janeiro, RJ, Brasil; 4 Departamento de Parasitologia, Universidade Federal de Minas Gerais – UFMG, Belo Horizonte, MG, Brasil; 5 Departamento de Parasitologia Animal, Universidade Federal Rural do Rio de Janeiro – UFRRJ, Seropédica, RJ, Brasil

**Keywords:** Haemulids, marine fish, helminths, parasitic crustaceans, community structure, parasite ecology, Haemulideos, peixe marinho, helmintos, crustáceos parasitos, estrutura da comunidade, ecologia parasitária

## Abstract

The barred grunt, *Conodon nobilis* (Linnaeus, 1758), is one of the most important marine-estuarine fish along the Brazilian coast. The present study evaluated the parasite fauna of this haemulid fish along the Southern Atlantic coast. From September 2010 to July 2011, a total of 100 specimens of *C*. *nobilis* from Angra dos Reis, Rio de Janeiro State, Brazil (23°01′21ʺS, 44°19′13ʺW), were examined. Ninety-seven individuals were parasitized by at least one species of metazoan, with a mean of 4.50 ± 3.54 parasites per fish. Eighteen species of parasites were collected: eight copepods, four digeneans, two cestodes, one acanthocephalan, one aspidogastrean, one isopod and one monogenean. The copepod *Lernanthropus rathbuni* was the most abundant and dominant species, accounting for 17.77% of all parasite specimens collected. *Caligus haemulonis* was the most prevalent. Prevalence and abundance of *Acantholochus lamellatus* and *L. rathbuni* tended to be higher in smaller fish, whereas those of *Torticaecum* sp. were higher in larger fish. The mean abundance of *C. haemulonis* was significantly higher in male hosts. A negative association was observed between two species of ectoparasites. The parasite community of *C. nobilis* was characterized by dominance of copepods, which can be related to host’s schooling behavior.

## Resumo

O roncador, *Conodon nobilis* (Linnaeus, 1758) é um dos peixes marinho-estuarinos mais importantes do litoral do Brasil. O presente estudo avaliou a fauna parasitária desse peixe, na costa sul do Atlântico. Entre setembro de 2010 a julho de 2011, um total de 100 espécimes de *C. nobilis*, da costa de Angra dos Reis, Rio de Janeiro, Brasil (23°01′21ʺS, 44°19′13ʺO), foram examinados. Noventa e sete indivíduos estavam parasitados por pelo menos uma espécie de metazoário, com média de 4,50 ± 3,54 parasitos por peixe. Dezoito espécies de parasitos foram coletadas: oito copépodes, quatro digenéticos, dois cestóides, um acantocéfalo, um aspidogastrídeo, um isópode e um monogenético. O copépode *L. rathbuni* foi a espécie mais abundante e dominante, representando 17,77% do total de parasitos coletados. *Caligus haemulonis* foi a espécie mais prevalente. A prevalência e abundância de *A*. *lamellatus*, *L*. *rathbuni* tenderam a ser maiores nos peixes menores, enquanto os valores de *Torticaecum* sp. maiores em peixes maiores. A abundância média de *C*. *haemulonis* foi significativamente maior em hospedeiros do sexo masculino. Associação negativa foi observada entre duas espécies de ectoparasitos. A comunidade parasitária de *C. nobilis* foi caracterizada pela dominância de copépodes, fato que pode estar relacionado ao comportamento de cardume do hospedeiro.

## Introduction

The Brazilian Atlantic coast extends for more than 8,000 km and supports about 1,240 fish species, of which 25 are haemulids (Euparcaria: Haemulidae) belonging to eight genera (Froese & Pauly, 2024). The coastal zone of the state of Rio de Janeiro is one of the largest in Brazil, with 636 km of extension. It forms an important aquatic ecosystem, composed of heterogeneous environments that contain highly diverse marine and estuarine organisms (Araújo et al., 2002; Kjerfve et al., 2021; Souza et al., 2021). However, with the expansion of urbanization and industrial development, anthropic activities have been causing diffuse pollution and a consequent decreased biodiversity off the coast of Rio de Janeiro (Araújo et al., 2017; Souza et al., 2021; Benicio et al., 2022).

Commonly known as grunts, haemulids are a typical group of actinopterygian fish, currently including 137 species from 21 genera, with broad global distribution, mainly inhabiting tropical and subtropical marine waters, while occasionally occurring in brackish water but rarely in freshwater (Nelson et al., 2016; Motta-Neto et al., 2019; Fricke et al., 2024). The genus *Conodon* Cuvier, 1830 is composed of only three species, all occurring in American marine ecosystems, of which *Conodon macrops* Hildebrand, 1946 and *Conodon serrifer* Jordan & Gilbert, 1882 are found in the Pacific Ocean (Froese & Pauly, 2024). The barred grunt *Conodon nobilis* (Linnaeus, 1758) is the only species occurring in the Atlantic Ocean, with distribution from the Caribbean (coast of Texas, eastern Florida and Jamaica) to Argentina (Froese & Pauly, 2024). Regarding biology, it is a demersal fish, found along sandy shores and over shallow muddy bottoms, feeding on crustaceans, mollusks and small fish (Menezes & Figueiredo, 1980; Froese & Pauly, 2024). In Brazilian waters, *C. nobilis* is not targeted by local fishers, although it has an important ecological role as a biotic vector, carrying organic matter from reefs to adjacent areas, as well as forming an important link in the coastal food web (Pombo et al., 2014; Da Silva et al., 2019; Motta-Neto et al., 2019).

Currently, more than 240 species of parasites have been recorded in haemulid fish in the coastal zone of North and South America, which indicates the high potential biodiversity of these organisms (Paschoal et al., 2015, 2022; López-Zacarías et al., 2021; García-Teh et al., 2022; Guillén-Hernández et al., 2023). In the South Atlantic Ocean, studies of the community of metazoan parasites of haemulids have been conducted only by Luque et al. (1996a, b), on the parasite fauna of *Orthopristis rubra* (Cuvier, 1830) and *Haemulon atlanticus* Carvalho, Marceniuk, Oliveira and Wosiacki, 2020 (reported as *H*. *steindachneri*), off Rio de Janeiro, Brazil. Recently, Paschoal et al. (2023) evaluated the parasite communities of three species of haemulid fish – *Anisotremus virginicus* (Linnaeus, 1758), *C. nobilis* and *O. rubra* – in the coastal waters of Rio de Janeiro, Brazil, using a comparative approach.

Based on the ecological importance of haemulid fish, specifically that of *C. nobilis*, in marine systems, and the scarcity of studies of the parasites of these organisms in Brazil, we analyzed the community structure of metazoan parasites of *C. nobilis*, in the coastal zone of Rio de Janeiro.

## Materials and Methods

### Host collection, processing and parasitological procedures

From September 2010 to July 2011, 100 specimens of *C. nobilis* were obtained along the coast of the city of Angra dos Reis (23°01′21ʺS, 44°19′13ʺW), state of Rio de Janeiro. The fish had been caught by local fishermen and were purchased at a local market. These were mostly fresh, but some were kept frozen at -20 °C until examination. The specimens were identified according to Menezes & Figueiredo (1980). The nomenclature and classification were updated according to FishBase (Froese & Pauly, 2024). Each fish was measured (total length), weighed and sexed. The Student *t*-test was used to verify possible differences in total length (data were normally distributed) between males and females, since parasite burdens can be correlated with host length (Zar, 2010).

Body surface, nostrils, gills, body cavities, esophagus, stomach, intestine, mesenteries, heart, liver, gonads, swim bladder and musculature of *C. nobilis* were individually examined for parasites, using a stereomicroscope. Parasite collection and processing were performed according to standard protocols (Eiras et al., 2006). Their identification followed the specific literature pertinent to each taxon.

### Ecological and statistical analyses

Prevalence, mean intensity and mean abundance were used as descriptors of the parasite population (at the component population level), according to Bush et al. (1997). The variance-to-mean ratio of parasite abundance (index of dispersion, ID) and the discrepancy index (D) were used to evaluate the distribution patterns of parasites in the host population (Poulin, 1993). The frequency of dominance (percentage of infracommunities in which a parasite species was numerically dominant) and the relative dominance (number of individuals of one species / total number of individuals of all species in the infracommunity) were calculated according to Rohde et al. (1995). 

The parasite diversity was calculated using the Brillouin index (*HB*), because theoretically each fish analyzed corresponded to a fully censused infracommunity (Magurran, 2004; Zar, 2010). Differences in parasite prevalence and abundance between male and female fish were evaluated using Fisher’s exact test (*F*) and the corrected normal approximation of the Mann-Whitney test (*Zc*), respectively (Magurran, 2004; Zar, 2010). Difference in parasite diversity according to host sex was also analyzed by the Mann-Whitney test (Magurran, 2004; Zar, 2010). Spearman’s rank correlation coefficient (*rs*) was used to verify the association (or its lack) of host length with parasite abundance, richness and diversity (Magurran, 2004; Zar, 2010). Host samples were also divided into three length intervals, namely: Class I - < 20.7 cm (immature), Class II - 20.8 cm – 27.9 cm (initial maturity); and Class III - > 28 cm (mature) (see Silva et al., 2019), in order to better evaluate some parasitological indicators according to fish maturity. Since the individuals of Class I were not parasitized, we excluded them from the analysis. Therefore, to test differences of parasite prevalence and abundance between host individuals from Class II and III, Fisher’s exact and Mann-Whitney tests were used, respectively, as previously indicated (Zar, 2010). To evaluate possible effects of concurrent infection (endoparasites) or infestation (ectoparasites), parasites were separated into three groups according to spatial guilds, as follows: adult endoparasites (digeneans infecting the stomach); helminth larval stages (digenean metacercariae and acanthocephalan cystacanths infecting the intestine); and adult ectoparasites (copepods infesting gills and operculum). Then, possible interspecific interferences involving the presence/absence of parasites were evaluated using logistic models, and possible association with interspecific parasite abundance was evaluated using Poisson regression models (Dohoo et al., 2003; Zar, 2010). 

The odds ratio (OR) of the statistically significant models (with 95% confidence interval [CI] for OR) was calculated in order to confirm its validity and evaluate the association between variables, in which 0 < OR < 1 indicates negative association, OR = 1 lack of association (invalid model) and OR > 1 denotes positive association (Dohoo et al., 2003). The ecological terminology follows that of Bush et al., (1997) and the analysis included only parasite species with prevalence higher than 10% (Bush et al., 1990). The statistical significance level was *p* ≤ 0.05. Statistical analyses were performed using the Quantitative Parasitology software (web version QPweb) (Reiczigel et al., 2019) and R software (R Core Team, 2020).

## Results

### Host samples

Fish mean total body length was 29.79 ± 5.14 (19.6-38.4) cm, and the mean weight was 430.13 ± 211.10 (94.5-880.7) g. The mean total body length of females was (28.92 ± 5.18, n = 56) and that of males was (30.91 ± 4.93, n = 44). The body lengths of males and females were not statistically different (*t* = 1.946, *p* = 0.629).

### Parasite component community

The parasite community of *C. nobilis* was composed of 18 species of metazoans (Table 1). The copepod *Lernanthropus rathbuni* Wilson, 1922 was the most abundant and dominant species, accounting for 17.77% of the total parasite specimens collected, with the highest mean relative dominance and frequency of dominance (Table 2). The copepod *Caligus haemulonis* Krøyer, 1863 was the most prevalent species (see Table 1).

**Table 1:** Metazoan parasites of *Conodon nobilis* associated with their parasitological descriptors, total number of individuals (TNI) and site of infection or infestation (Site), in the coast off Rio de Janeiro, Brazil.

**Parasite species**	**Parasitological descriptors**			**TNI**	**Site**
	**P (%)**	**MI ± SD**	**MA ± SD**		
**Digenea**					
*Aponurus laguncula*	27	2.03 ± 1.37	0.55 ± 1.14	55	Stomach
*Genolopa ampullacea*	5	2.21 ± 0.83	0.11 ± 0.51	11	Stomach
*Parahemiurus merus*	13	2.07 ± 1.55	0.27 ± 0.88	27	Stomach
*Torticaecum* sp. (matacercariae)	23	1.95 ± 1.58	0.45 ± 1.11	45	Intestine
**Aspidogastrea**					
*Lobatostoma ringens*	2	4	0.08 ± 0.56	08	Intestine
**Cestoda**					
*Calliterarhynchus* sp. (plerocercoid)	7	1.14 ± 0.37	0.08 ± 0.30	08	Mesentery
*Pterobothrium* sp. (plerocercoid)	4	1	0.04 ± 0.19	04	Mesentery
**Monogenea**					
*Encotyllabe spari*	6	1	0.06 ± 0.23	06	Pharynx
**Acanthocephala**					
*Serrassentis* sp. (cystacanth)	12	1.16 ± 0.38	0.14 ± 0.40	14	Intestine
**Copepoda**					
*Acantholochus lamellatus*	32	2.03 ± 1.25	0.65 ± 1.18	65	Gills
*Caligus haemulonis*	39	1.89 ± 1.42	0.74 ± 1.28	74	Operculum
*Caligus longipedis*	1	1	0.01	01	Operculum
*Caligus robustus*	10	1.2 ± 0.42	0.12 ± 0.38	12	Operculum
*Caligus rufimaculatus*	2	1	0.02	02	Operculum
*Caligus xystercus*	2	1.5 ± 0.70	0.03 ± 0.22	03	Operculum
*Hamaticolax* sp.	1	1	0.02	02	Nostril
*Lernanthropus rathbuni*	37	2.16 ± 1.80	0.81 ± 1.51	80	Gills
**Isopoda**					
*Gnathia* sp. (larval)	8	4.12 ± 3.56	0.33 ± 1.47	33	Operculum

P: prevalence; MI: mean intensity; MA: mean abundance; SD: standard deviation.

**Table 2:** Frequency of dominance and mean relative dominance of metazoan parasites of *Conodon nobilis* from the coast off Rio de Janeiro, Brazil.

**Parasite species**	**Frequency of dominance**	**Frequency of dominance shared with one or more species**	**Mean relative dominance**
*Lernanthropus rathbuni*	17	8	0.179 ± 0.343
*Caligus haemulonis*	13	9	0.129 ± 0.272
*Acantholochus lamellatus*	10	10	0.110 ± 0.240
*Aponurus laguncula*	7	11	0.081 ± 0.184
*Torticaecum* sp. (metacercariae)	7	5	0.071 ± 0.214
*Parahemiurus merus*	4	4	0.036 ± 0.142
*Serrassentis* sp. (cystacanth)	1	3	0.020 ± 0.117

Adult ectoparasites corresponded to 54.44% of all parasite specimens collected, adult endoparasites to 22.44%, larval endoparasites to 15.77% and larval ectoparasites to 7.33%. All parasites of *C. nobilis* showed the typical aggregate distribution, in which ID and D values were higher than 1.46 and 0.70, respectively (Table 3). The abundance of the copepods *L. rathbuni* and *Acantholochus lamellatus* Paschoal, Cezar and Luque, 2013 was negatively correlated with host total body length (*rs* = -0.23, *p* = 0.02; *rs* = -0.20, *p* = 0.04, respectively), and their abundance (*p* = 0.007 and *p =* 0.001, respectively) and prevalence (*p* = 0.003 and *p* = 0.002, respectively) were higher in hosts from Class II (Table 4). Regarding endoparasites, the abundance of the digenean *Torticaecum* sp. was positively correlated with host total length (*rs* = 0.21, *p* = 0.03), and its abundance (*p* = 0.025) and prevalence (*p =* 0.005) were higher in fish from Class III. The digenean *Parahemiurus merus* (Linton, 1910) was also more prevalent in hosts from Class III (*p =* 0.040) (Table 4). The mean abundance of *C*. *haemulonis* was significantly higher in male (1.068 ± 1.065) than in female (0.482 ± 0.894) hosts (*Zc=* 2.23, *p* = 0.025).

**Table 3:** Measurements for parasite dispersion within host population, indicated by variance to mean ratio (ID) and index of Discrepancy (D) of metazoan parasites of *Conodon nobilis*, from the coast off Rio de Janeiro, Brazil.

**Parasite species**	**ID**	**D**	**Type of dispersion**
*Acantholochus lamellatus*	2.16	0.774	Aggregate
*Aponurus laguncula*	2.41	0.808	Aggregate
*Caligus haemulonis*	2.23	0.737	Aggregate
*Lernanthropus rathbuni*	2.85	0.756	Aggregate
*Parahemiurus merus*	2.91	0.907	Aggregate
*Serrassentis* sp. (cystacanth)	1.46	0.885	Aggregate
*Torticaecum* sp. (metacercariae)	2.76	0.841	Aggregate

**Table 4:** Prevalence and mean abundance of metazoan parasites of *Conodon nobilis* from the coast off Rio de Janeiro, Brazil, associated with hosts body length classes.

**Parasite species**	**Class I (n=03)**		**Class II (n=30)**		**Class III (n=67)**	
	**P(%)**	**MA**	**P(%)**	**MA ± SD**	**P(%)**	**MA ± SD**
*Lernanthropus rathbuni*	-	-	60*	1.66 ± 2.29**	28.3*	0.44 ± 0.78**
*Acantholochus lamellatus*	-	-	60*	1.23 ± 1.41**	20.8*	0.41 ± 1**
*Caligus haemulonis*	-	-	36.6	0.53 ± 0.89	41.7	0.86 ± 1.43
*Aponurus laguncula*	-	-	26.6	0.43 ± 0.81	28.3	0.62 ± 1.28
*Torticaecum* sp. (metacercariae)	-	-	6.6*	0.10 ± 0.40**	31.3^*^	0.62 ± 1.30^**^
*Serrassentis* sp. (cystacanth)	-	-	6.6	0.06 ± 0.25	14.9	0.17 ± 0.45
*Parahemiurus merus*	-	-	3.3*	0.03 ± 0.18	17.9*	0.38 ± 1.05

P: prevalence; MA: mean abundance; SD: standard deviation; significant differences of the parasite prevalence* and abundance** among the host length classes.

### Parasite infracommunities

Ninety-seven specimens (97%) of *C. nobilis* were parasitized by at least one taxon of metazoan. A total of 450 parasite specimens were collected, with a mean of 4.50 ± 3.54 per host. The total body length of fish and parasite abundance were not correlated (*rs* = 0.027, *p* = 0.782). The mean species richness of parasites was 2.32 ± 1.25, and also was not correlated with host total body length (*rs* = 0.053, p = 0.595). Similarly, mean parasite diversity (*HB* = 0.411 ± 0.336) was not correlated with host body length (*rs* = 0.103, p = 0.306), and no significant differences regarding this descriptor were observed between male (*HB* = 0.418 ± 0.351) and female (*HB* = 0.406 ± 0.327) hosts (*Zc* = 0.277, *p* = 0.390). Twenty-eight host specimens (28%) were infected/infested by a single parasite species; 25 (25%) by two, 29 (29%) by three, 9 (9%) by four, 5 (5%) by five and 1 (1%) by six species.

Interspecific associations among the adult digeneans were possible to evaluate only between the species *Aponurus laguncula* Looss, 1907 and *P*. *merus*. However, no apparent interaction was observed, since their presence/absence and their abundances were not correlated according to the statistical models (*p* = 0.104 and *p* = 0.743, respectively). Similar results were observed in concurrent infections by larval forms of *Torticaecum* sp. and *Serrassentis* sp. (*p* = 0.861 and *p* = 0.318, respectively). Concurrent infestations by copepods were observed among three species, *A*. *lamellatus*, *C*. *haemulonis* and *L*. *rathbuni*, which occurred in permuted pairs. However, the only possible interspecific interaction was observed between *C*. *haemulonis* and *L*. *rathbuni*, in which the abundances showed negative association (*p* = 0.036; OR = 0.766; CI = 0.597–0.983).

## Discussion

Previous studies of the parasite fauna of *C*. *nobilis* have reported 23 taxa (larvae and adults) in the Atlantic Ocean, including Copepoda (10), Digenea (6), Cestoda (2), Acantocephala (1), Aspidogastrea (1), Isopoda (1), Monogenea (1) and Nematoda (1), of which 20 (87%) of these taxa have been reported in Brazil, 2 (8.6%) in the Bahamas and 1 (4.3%) in Puerto Rico (Paschoal et al., 2015, 2023). All parasites found in the present study have been recorded parasitizing *C*. *nobilis*, but no previous study has investigated its parasite community structure or any other congener. Therefore, these results indicate *C*. *nobilis* has a highly diverse parasite fauna and is a relevant target for investigation to better comprehend these organisms, especially regarding host-parasite and parasite-parasite interactions.

Parasitic copepods were dominant in the present parasite community of *C. nobilis*. The dominance by ectoparasites has been observed in only a few parasite communities of marine fish from the South Atlantic Ocean, and probably is favored by the crowding behavior of these fish in high population densities, which facilitates the transmission of ectoparasites with direct life cycles, such as copepods (Cezar & Luque, 1999; Tavares et al., 2001; Tavares & Luque, 2004; Alves & Luque, 2006). Haemulids commonly occur in large shoals that may include different species of coral reef fish, e.g., belonging to the Carangidae, Lutjanidae and Scaridae families (Pereira et al., 2011; Cerqueira et al., 2021). Moreover, according to Pombo et al. (2014), *C. nobilis* has high population density in southeastern Brazil. The copepods found in the present study were represented by species that have been reported on haemulids (*C*. *haemulonis, Caligus xystercus* Cressey, 1991 and *L. rathbuni*), carangids (*Caligus longipedis* Bassett-Smith, 1898, *Caligus rufimaculatus* Wilson, 1905 and *Caligus robustus* Bassett-Smith, 1898) and exclusively on *C*. *nobilis* (*A. lamellatus*) (Luque & Tavares, 2007; Paschoal et al., 2013). Therefore, it is plausible that the high population density of *C*. *nobilis* associated with mixed shoals favors the transmission of copepods in the ecosystem evaluated by us.

The copepod *L*. *rathbuni* was the most abundant and dominant species in the parasite community of *C. nobilis*. Currently, about 17 species of lernanthropids have been reported, infesting more than 20 species of marine fish from the Brazilian coast (Luque & Tavares, 2007; Luque et al., 2013). Of these, *L*. *rathbuni* is the most commonly recorded and infests five species of haemulids, namely, *Anisotremus surinamensis* (Bloch, 1791), *C. nobilis*, *H*. *atlanticus*, *Haemulopsis corvinaeformis* (Steindachner, 1868) and *O*. *rubra* (Paschoal et al., 2015, 2023). In the North Atlantic Ocean, *L*. *rathbuni* has also been reported parasitizing the haemulid fish *Orthopristis chrysoptera* (Linnaeus, 1766) along the coast of Florida, USA (Paschoal et al., 2015). Host preference exhibited by parasitic copepods is not uncommon, being a consequence of the capacity of these organisms to locate, identify and establish themselves on their hosts (Mordue & Birkett, 2009). The sea louse *Lepeophtheirus salmonis* (Kroyer, 1837), for example, infests more than 10 species of salmonid fish in the Northern Hemisphere, using phototropism, chemotropism, and mechanical perception to reach its hosts (Mordue & Birkett, 2009). The host range of *L*. *rathbuni* in the Atlantic Ocean, composed by certain species of haemulid fish, can possibly be explained by mechanisms similar to those previously described. However, the host recognition mechanisms used by *L*. *rathbuni* are still unknown, since no study of the biology of this species has been published. It should be mentioned that, despite the affinities between *L. rathbuni* and haemulid fish, its prevalence varies in the South Atlantic Ocean, ranging from 18% in infestations of *H*. *atlanticus*, *H. corvinaeformis* and *O*. *rubra*, to 37% in infestations of *C. nobilis* (Luque et al., 1996a; Cavalcanti et al., 2006), illustrating the host selection by *L*. *rathbuni* even among haemulid fish.

Parasite community structure in fish has been related to a series of host factors, such as biology, body size, aggregate distribution (shoaling behavior), hormonal variations and ventilation volume during gas exchange/hemostasis (Boxshall & Halsey, 2004; Poulin et al., 1991; Timi & Lanfranchi, 2006). The influence of host body size on the prevalence and abundance of parasitic copepods has been observed in several natural associations with fish, in which some authors have suggested that increased body and gill surfaces along with higher ventilation volume are key factors influencing these parasitological descriptors (Dogiel et al., 1961; Hanek & Fernando, 1978; Kabata, 1982; Cressey et al., 1983; Timi & Lanfranchi, 2006). Cressey et al. (1983) observed that infestation patterns of *Pseudocycnoides* spp. on scombrid fish were influenced by the ability of these copepods to adhere to gill filaments. These authors observed that for effective attachment, the appendages of these copepods should partially embrace the gill filament, which was facilitated in hosts measuring 20 to 30 cm. In the present study, the most adequate body size range of *C*. *nobilis* for infestations by *L*. *rathbuni* and *A*. *lamellatus* was 20.8 to 27.9 cm, defined as Class II, since both prevalence and abundance of these parasites were higher in hosts of this class (see Table 4). Moreover, the abundances of these copepods were negatively correlated with host body length, as a consequence of the previously described results. Those observations suggest that the size of gill filaments of *C*. *nobilis* allocated in Classes I (too small) and III (too large) is not as suitable for the attachment to the appendages of *L*. *rathbuni* and *A*. *lamellatus* as are the gill filaments of those fish allocated in Class II, similar to the findings of Cressey et al. (1983).

Changes in endoparasite burdens in fish hosts have been associated with variations in body size and are commonly explained by ontogenetic changes in diet composition and spatial availability within the host organism (Polyanski, 1961; Saad-Fares & Combes, 1992; Sabas & Luque, 2003). Details of the life cycle of most species of digeneans that parasitize fish are unavailable, but it is well known that mollusks are crucial, acting as first intermediate obligatory hosts (Cribb et al., 2001, 2003). The diet of *C. nobilis* is mainly composed of invertebrates and small bony fish (Lopes & Oliveira-Silva, 1998; Feitosa et al., 2002). Pombo et al. (2014) identified a total of seven prey items in the diet of *C. nobilis*, in which there was preference for crustaceans of the orders Mysida Boas‎, 1883‎ and Amphipoda Latreille‎, 1816‎, while cnidarians, mollusks and polychaetes were rarely consumed. In the present study, the prevalence and abundance of *Torticaecum* sp. (metacercariae) were higher in hosts from Class III (the largest), and its abundance was positively correlated with host fish body length. These results are possibly related to cumulative infection, in which the consumption of potential intermediate hosts of this digenean (e.g., mollusks) increases proportionally to fish growth. Such cumulative effect is common in helminth larval forms and similar results have been observed in other marine fish from the South Atlantic Ocean (Luque et al., 1996a; Knoff et al., 1997; Luque & Chaves, 1999; Alves & Luque, 2001; Sabas & Luque, 2003). It should be mentioned that although no statistical differences were observed in the prevalence and abundance of *P*. *merus* between hosts from Class II and III, the values of these parasitological descriptors were higher in hosts of Class III. This can also be also explained by the higher consumption of potential intermediate hosts by larger fish, as well as by the fact that in larger hosts, more space is available for parasite colonization.

According to Lizama et al. (2005), fish sex can influence the host-parasite relationships. In the present study, the absence of difference in body length according to sex of *C*. *nobilis* was common, since both males and females reach sexual (gonadal) maturity at similar body lengths, as demonstrated by a previous study (da Silva et al., 2019). The fact that the abundance of C. *haemulonis* was higher in males of *C. nobilis* may be associated with the relative scarcity of behavioral and physiological differences related to sex. Moreover, such differences of infestation by *C. haemulonis* between male and female fish observed here most likely is explained by casual factors that commonly influence ectoparasite loads, such as seasonal changes in the composition of the tegument; high testosterone levels, which can cause immunosuppression in males; and/or behavioral differences between sexes (Pickering, 1977; Folstad & Karter, 1992; Poulin, 1996; Tavares & Luque, 2004; Lizama et al., 2005). Associations between host sex and ectoparasite infestations in wild fish populations have been documented in the South Atlantic Ocean (see Luque et al., 1996a; Knoff et al., 1997; Chaves & Luque, 1999; Alves & Luque, 2001).

Regarding parasite species richness (at the component community level) of haemulids studied so far in the South Atlantic Ocean, the parasite community of *O*. *rubra* studied by Luque et al. (1996a, b) contained 21 different species, contrasting with that of *C. nobilis* in the present study, which was composed of 18 species. The parasite community of *H*. *atlanticus*, also studied by Luque et al. (1996a, b), was composed of 18 species, and that of *A*. *virgincus* was composed of 16 parasite species (see Paschoal et al., 2023). According to some authors, the higher richness observed in the parasite component community of *O*. *rubra* may be related to its broader vagility, greater dietary diversity (related to endoparasite infections), and anatomical and physiological particularities (Luque et al., 1996b, 2004; Paschoal et al., 2023). These parasite communities of haemulid fish off the Brazilian coast are all composed by ecto and endoparasites, in which the latter are represented by adult and larval forms (Luque et al., 1996b, 2004; Paschoal et al., 2023). These findings indicate that such fish occupy intermediate positions within the local trophic webs, acting as both intermediate and definitive hosts. Therefore, these haemulids can be considered important trophic links in the ecosystems. It should be mentioned that their parasite communities show varied species composition and particular structures according to the host species. For example, the acanthocephalan cystacanth *Serrassentis* sp. has been reported in *A*. *virgincus*, *C. nobilis*, *H*. *atlanticus* and *O*. *rubra*, whereas the copepods *C*. *haemulonis* and *L*. *rathbuni* have been reported infesting *C*. *nobilis*, *H*. *atlanticus* and *O*. *rubra*, in which parasite burdens vary according to the fish species (Paschoal et al., 2023). In any case, it is important to highlight that knowledge pertaining to parasites of haemulid fish from the South Atlantic Ocean is still incipient, preventing further conclusions on patterns of parasite species richness, since only four species of haemulids, out of 25 that occur in this region, have been studied for parasites (Luque et al., 1996a, b; Paschoal et al., 2023).

Positive or negative associations between parasite species can provide evidence of interspecific interactions and the community structure (Poulin, 2001; Benicio et al., 2022). The low number of concurrent infections/infestations observed in the present study was similar to the results obtained by Luque et al. (1996b), regarding the parasites of *H*. *atlanticus*, from the same location as that of the present study. Such findings indicate the isolationist characteristic of these parasite communities (see Luque et al., 1996b). In contrast, the parasite community of *O. rubra* seems to be more interactive, since concurrent infections were more frequent and there was evidence of interspecific interactions, as also reported by Luque et al. (1996b) in the same area that we studied. According to Poulin (2001), changes in parasite infrapopulation structure, as indicated by mean abundance or prevalence, for example, may be evidence of interaction in cases of interspecific co-occurrence. On the other hand, the literature suggests that interspecific interactions among parasites can only be validated under experimental conditions (see Rohde et al., 1995; Poulin, 2001). The higher interactive nature observed in the parasite community of *O*. *rubra* may be a consequence of higher species richness and much higher number of individual parasites (7,282) (see Luque et al., 1996b) in comparison with the parasite communities of the other haemulid species cited previously. It is logical to expect a greater number of species and individuals in a parasite community to be associated with a higher probability of interaction among them. Nevertheless, despite the number of concurrent infections between endoparasites, and infestations between copepod ectoparasites, the only possible indication of interspecific interaction was observed in concurrent infestations by C. *haemulonis* and *L*. *rathbuni*, in which their abundances were negatively associated. These species occupy different, but closely related, sites in the host (operculum and gill, see Table 1), placing them physically close together. However, the cause of such interference cannot be established based on the present results, since more detailed data would be required. The discussion in this paragraph only reinforces the premise that parasite communities of marine fish are stochastic and very complex, making it difficult to define general patterns (Holmes, 1990; Rohde et al., 1995; Tavares & Luque, 2004).
